# The function of chemokine-driven glial-neuronal interaction in chronic pain

**DOI:** 10.3389/fnins.2026.1830053

**Published:** 2026-05-01

**Authors:** Mingyuan Fan, Zhi Liang, Jiayi Shen, Hui Yang, Liping Zhao, Min Li, Jie Zheng

**Affiliations:** 1 College of Acupuncture and Tuina, Shaanxi University of Traditional Chinese Medicine, Xianyang, Shaanxi, China; 2 Department of the Second Affiliated Hospital of Shaanxi University of Traditional Chinese Medicine, Xianyang, Shaanxi, China

**Keywords:** chemokines, chronic pain, glial cells, neuroinflammation, neurons

## Abstract

Chronic pain affects hundreds of millions worldwide, yet current treatments—from NSAIDs to opioids—fall short and bring unwanted side effects. Neuroinflammation drives bidirectional glial-neuronal communication, a core mechanism underlying central sensitization in chronic pain, with chemokines serving as key mediators. Most studies to date have zeroed in on isolated pathways or specific anatomical sites, missing the bigger picture. We pull together findings across the neural axis, spanning peripheral sensory ganglia, the spinal dorsal horn, and higher brain centers, then dissect the molecular machinery of chemokine-mediated crosstalk among microglia, astrocytes, and neurons. We trace downstream regulatory cascades through two major axes—CCL2/CCR2 and CX3CL1/CX3CR1—along with their effector branches (ERK-GluN2B, p38 MAPK-NF-κB), map out polarization phenotypes (M1/M2 for microglia, A1/A2 for astrocytes), and detail the functional shifts glia undergo in disease states. By knitting scattered observations into a unified regulatory map of chemokine signaling across the chronic pain neural network, this review addresses the field’s persistent lack of systematic integration.

## 1 Introduction

Chronic pain—marked by spontaneous pain, hyperalgesia, and dysesthesia persisting three months or longer—remains a formidable clinical challenge. Sustained noxious exposure lowers pain receptor thresholds, amplifying responses to stimuli (Corder et al., 2019). Patients frequently grapple with despair, depression, and anxiety; in severe cases this escalates to substance abuse or suicide (Paul et al., 2025). In China alone, prevalence hovers around 35.9%, imposing heavy economic burdens on individuals and society (Han et al., 2025). Frontline therapies still center on NSAIDs and opioids, yet these agents lack specificity and often provide limited relief (Kohno and Tsuda, 2025). Mapping chronic pain pathophysiology and designing targeted interventions has become an urgent priority for neuroscience and public health.

The neurogenic inflammation hypothesis offers a useful framework: physical injury, neurodegeneration, autoimmune assault, or toxins can ignite localized inflammatory cascades in the peripheral or central nervous system (Gilhus and Deuschl, 2019). Neurons were once viewed as the sole actors in pain transmission, but the past decade has redrawn this picture. Glial cells—microglia and astrocytes in particular—are now recognized as critical drivers of chronic pain onset and maintenance, with bidirectional glial-neuronal communication serving as the engine behind central sensitization (Donnelly et al., 2020). These processes coincide with glial hyperactivation, disrupted synaptic plasticity, and broad inflammatory escalation (McKenzie et al., 2025).

Chemokines and their receptors have surfaced as key mediators of neuron-glial crosstalk. Recent studies show these molecules actively shape chronic pain by triggering glial activation and modulating neuronal sensitization, effectively bridging central and peripheral mechanisms (Lyu et al., 2021; Luo et al., 2016). Chemokines are small cytokines (roughly 8–15 kDa) comprising approximately 50 family members that bind specific cell surface receptors (Wyse et al., 2017; Colobran et al., 2007; Do et al., 2020; Dillenburg-Pilla et al., 2015). Beyond their classical role in leukocyte chemotaxis, chemokines have emerged as pivotal messengers in aberrant neuron-glial communication, placing them at the forefront of chronic pain research.

Understanding the mechanisms of chemokines has accelerated the process of clinical translation. For example, a Phase II trial of the CCR2/CCR5 antagonist CNTX-6970 for knee osteoarthritis demonstrated that the antagonist has an acceptable safety profile (Edwards et al., 2026); the multi-target antagonist RAP-103 has entered Phase II clinical development (Ruff et al., 2022), and its allosteric modulators (proteoglycans targeting CCR2) are also emerging (Park et al., 2022). These research advances highlight the need to further optimize chemokine-targeted therapeutic strategies.

Research on specific axes—notably CCL2/CCR2 and CX3CL1/CX3CR1—has advanced considerably (Bai et al., 2023). Yet the literature remains fragmented, with studies scattered across individual pathways or anatomical regions and little integration across the neural hierarchy, from peripheral ganglia and spinal dorsal horn up to cortical and hypothalamic centers. Meanwhile, fresh data continue to illuminate how chemokine-driven glial-neuronal interactions influence pain sensitization, emotional disturbances, and homeostatic dysregulation. The field urgently needs a synthesis that knits these disparate advances into a coherent picture of chemokine signaling logic across chronic pain networks.

This review addresses that need. From the cytokine vantage point, we examine how chemokines orchestrate glial-neuronal interactions across neuroanatomical territories to govern chronic pain initiation and persistence. By synthesizing recent evidence spanning peripheral processing, ascending transmission, cortical emotional encoding, descending modulation, and homeostatic control, we aim to sketch a more complete portrait of chronic pain pathophysiology and identify therapeutic targets for clinical translation.

## 2 Glial-mediated neuroimmune responses contribute to the chronicity of pain

Glial cells lie at the heart of persistent pain. In the central nervous system, they outnumber neurons by an estimated 10–50 times. The majority are astrocytes, microglia, and oligodendrocytes—astrocytes alone account for 20–40% of the total (Ji et al., 2016; Clark et al., 2007). Peripheral nerve injury activates both peripheral immune cells and glial cells at multiple sites. Once activated, glia release pain mediators; this aberrant activation triggers events that amplify and prolong pain signals.

### 2.1 Microglia

Microglia, the CNS resident immune cells, modulate neuronal activity and shape synapse formation and transmission. They support neuronal proliferation and differentiation while providing protection and nourishment (Wang et al., 2024), placing them squarely in pain pathophysiology (Chen G. et al., 2018). Fate mapping with Runx1cre mice traces microglial origins to yolk sac hemangioblasts (EMP) (Zhao et al., 2023). Their core functions include physical engagement with synapses, debris, and extracellular matrix during phagocytosis, plus neurotransmitter secretion (Parkhurst et al., 2013; Shigemoto-Mogami et al., 2014).

Pathological conditions trigger morphological transformation: microglia shift from their typical ramified shape—long, branching processes—to an amoeboid form with stubby processes and swollen cell bodies, signaling functional activation (Vidal-Itriago et al., 2022). These cells have been implicated in chronic pain pathophysiology (Inoue and Tsuda, 2018). Activated by external or internal cues, microglia can polarize into two distinct phenotypes when activated by extracellular or intracellular cues: the pro-inflammatory M1 phenotype and the anti-inflammatory M2 phenotype (Table 1).

**Table 1:** Comparative characteristics of microglial M1 and M2 phenotypes in chronic pain.

Feature	M1 phenotype	M2 phenotype
Inducing stimuli	LPS, IFN-*γ*, TNF-α, IL-1β	IL-4, IL-13, IL-10
Surface markers	CD86, CD16/32, MHC-II	CD206, CD163
Functional enzymes	iNOS↑, ROS↑	Arginase-1↑, Ym1/2↑
Secreted mediators	IL-6, IL-23, IL-1β, TNF-α, CCL2, CXCL10	BDNF, IGF-1, TGF-β
Core functions	Pro-inflammatory response; neuroinflammation amplification; neuronal hyperexcitability	Phagocytosis of debris; tissue repair; neuroprotection; inflammation resolution
Role in chronic pain	Perpetuates pain sensitization	Facilitates pain resolution

BDNF, brain-derived neurotrophic factor; IFN-γ, interferon-γ; IGF-1, insulin-like growth factor-1; IL, interleukin; iNOS, inducible nitric oxide synthase; LPS, lipopolysaccharide; MHC-II, major histocompatibility complex class II; ROS, reactive oxygen species; TGF-β, transforming growth factor-β; TNF-α, tumor necrosis factor-α.

Nerve injury triggers synaptic remodeling in the S1 cortex and drives pyramidal neuron hyperexcitability; selectively knocking out BDNF in microglia eases these changes, and the same BDNF deficiency in S1 microglia reduces pain behaviors after peripheral nerve injury (Huang et al., 2021). These observations point to microglia tuning pain-related neuroplasticity via released signaling molecules like BDNF.

### 2.2 Astrocytes

Current research on astrocyte-mediated pain predominantly concentrates on the spinal cord level. Astrocyte activation is typically indicated by elevated production of glial fibrillary acidic protein (GFAP) or by astrocytic proliferation (Ben Haim and Rowitch, 2017). Recent research has revealed two unique categories of reactive astrocytes: A1-reactive astrocytes and A2-reactive astrocytes (Liddelow et al., 2017). These distinct phenotypes exhibit contrasting roles in neuroinflammation and chronic pain pathophysiology (Table 2).

**Table 2:** Comparative characteristics of astrocytic A1 and A2 phenotypes in chronic pain.

Feature	A1 phenotype	A2 phenotype
Inducing stimuli	Microglia-derived IL-1α, TNF-α, C1q	Ischemia, tissue damage, anti-inflammatory signals
Molecular markers	C3, C1q, Serping1, Lcn2↑	BDNF, GDNF, FGF, Spp1, Thbs1/2↑
Functional changes	Loss of supportive functions; reduced glutamate uptake; impaired metabolic support	Enhanced phagocytosis; synapse formation promotion; metabolic support preserved
Effect on neurons	Neurotoxic; induces neuronal and oligodendrocyte death	Neuroprotective; supports neuronal survival
Core functions	Pro-inflammatory; exacerbates neuroinflammation	Tissue healing; functional recovery; inflammation dampening
Role in chronic pain	Maintains pro-nociceptive environment; disrupts synaptic homeostasis	Promotes pain resolution; restores homeostasis

BDNF, brain-derived neurotrophic factor; C1q, complement component 1q; C3, complement component 3; FGF, fibroblast growth factor; GDNF, glial cell line-derived neurotrophic factor; IL-1α, interleukin-1α; Lcn2, lipocalin-2; Serping1, serpin family G member 1; Spp1, secreted phosphoprotein 1 (osteopontin); Thbs, thrombospondin; TNF-α, tumor necrosis factor-α.

Astrocytes modulate pain through three functional routes (Ji et al., 2014; Lu and Gao, 2023): shifts in glial signaling pathways, including altered MAPK phosphorylation and transcription factor expression; dysregulation of receptor and channel proteins, marked by upregulated inflammatory factor receptors and gap junction proteins alongside downregulated glutamate transporters; and persistent release of glial mediators spanning chemokines, cytokines, and proteases.

### 2.3 Interactions among glia, neurons, and the immune system in chronic pain

Crosstalk among astrocytes, microglia, and neurons is essential for neural function regulation (Allen and Lyons, 2018). Spinal and brain glial-neuronal interactions can drive central sensitization—heightened neuronal responses to normal or subthreshold inputs. Astrocyte-microglia communication depends on secreted factors: growth factors, neurotransmitters, cytokines, and chemokines involved in metabolism and tissue change (Haydon, 2001).

Astrocytes communicate directly with neurons, not just microglia. In the SNL model, CXCL13 and its receptor CXCR5 spike in neurons and glial cells; knock out Cxcr5, and the glial response to nerve injury drops dramatically (Jiang et al., 2016). Moreover, CCL2 and CXCL1 released by activated astrocytes can stimulate CCR2 and CXCR2 receptors on spinal neurons, boosting excitatory synaptic transmission and yielding chronic pain (Jiang et al., 2020). The data suggest these three cell types form a feedback loop that cooperatively modulates chronic pain.

## 3 Chemokines: key mediators of glial-neuronal communication in chronic pain

### 3.1 Overview of chemokines and their receptors

Chemokines are small cytokines (about 8–15 kDa) with a characteristic N-terminal cysteine residue, sorted into four subfamilies (Solis-Castro et al., 2021; Old and Malcangio, 2012): CC, CXC, XC, and CX3C. As potent mediators of neuron-glial crosstalk in pathological pain, chemokines form a distinct cytokine group with approximately 50 members, widespread in blood and immune tissues (Solis-Castro et al., 2021). They also populate the CNS, especially astrocytes (Kiguchi et al., 2012). By engaging G protein-coupled receptors (GPCRs), chemokines spark downstream signaling cascades that shape chronic pain development and maintenance (Wang et al., 2024). Among the many chemokine-receptor pairs, CCL2/CCR2 and CX3CL1/CX3CR1 stand out as the most extensively studied pathways in chronic pain research, given their central role in neuroimmune interactions (Ciechanowska and Mika, 2024).

### 3.2 Mechanisms of action of the CCL2/CCR2 signaling axis in chronic pain

#### 3.2.1 Molecular characteristics and cellular distribution of the CCL2/CCR2 axis

CCL2 (monocyte chemotactic protein-1, MCP-1) represents the CC subfamily; its specific receptor CCR2 appears mainly on peripheral macrophages, monocytes, and spinal dorsal horn neurons (Gosselin et al., 2005; Ji et al., 2013). In neuropathic and inflammatory pain models, CCL2 surges in DRG neurons and spinal dorsal horn astrocytes (Zhang et al., 2017; Ryu et al., 2024; Xie et al., 2018). Recent work shows CCR2 is functionally expressed on excitatory glutamatergic neurons (VGLUT2-positive) in spinal lamina II, furnishing the structural basis for CCL2 direct regulation of central sensitization (Zhang et al., 2020a).

#### 3.2.2 Downstream signaling cascades mediated by the CCL2/CCR2 axis in central sensitization

**Figure 1:**
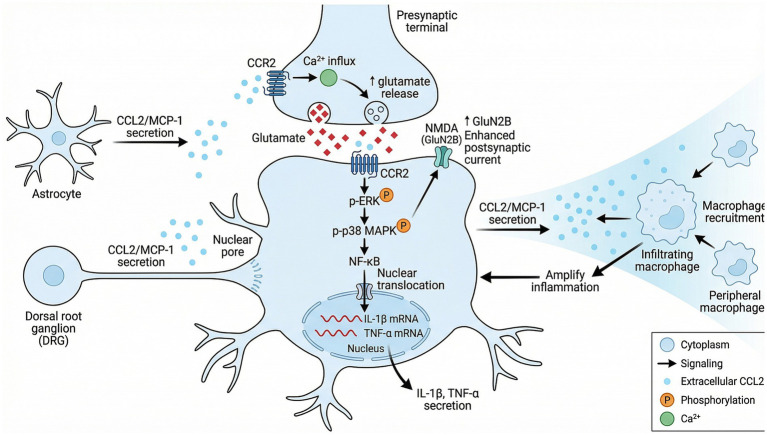
Schematic illustration of CCL2/CCR2-mediated neuroinflammation and central sensitization. This diagram depicts the multi-cellular regulatory mechanism of the C-C motif chemokine ligand 2 (CCL2, also known as monocyte chemoattractant protein-1, MCP-1)/C-C chemokine receptor type 2 (CCR2) signaling axis in the spinal dorsal horn. Astrocytes and dorsal root ganglion (DRG) neurons secrete CCL2, which acts on presynaptic terminals to trigger Ca^2+^ influx and enhance glutamate release. Postsynaptically, CCL2 binds to neuronal CCR2, inducing phosphorylation of extracellular signal-regulated kinase (ERK) and p38 mitogen-activated protein kinase (MAPK), which further promotes nuclear translocation of nuclear factor kappa-B (NF-κB). This leads to the transcription and secretion of pro-inflammatory cytokines interleukin-1 beta (IL-1*β*) and tumor necrosis factor-alpha (TNF-*α*). Meanwhile, this signaling upregulates the GluN2B subunit of N-methyl-D-aspartate (NMDA) receptors, enhancing postsynaptic currents to mediate central sensitization. Additionally, neuronal CCL2 recruits peripheral macrophages, which in turn secrete more CCL2 to amplify local inflammation, forming a positive feedback loop.

The distribution pattern described above shows CCL2/CCR2 can orchestrate central sensitization through multiple mechanisms. Specifically, this axis assembles a regulatory cascade spanning postsynaptic, transcriptional, and presynaptic levels that collectively push neuronal excitability upward (Figure 1).

1. ERK-GluN2B signaling pathway

At the postsynaptic level, CCL2 rapidly triggers intracellular signaling via G protein-coupled mechanisms upon binding CCR2. Zhang et al. (2020a) used patch-clamp plus single-cell PCR to show CCL2 rapidly enhances NMDA receptor-mediated currents in spinal lamina II neurons through the CCR2-ERK pathway; this effect flows mainly through NMDA receptors containing the GluN2B subunit. The mechanism runs as follows: CCL2 activates CCR2, which phosphorylates ERK through the downstream Ras–Raf–MEK cascade; pERK then ramps up GluN2B subunit expression on neuronal surfaces and boosts its functional activity (Lu et al., 2022). The GluN2B-specific blocker ifenprodil completely shuts down CCL2 potentiation of NMDA currents and markedly relieves CFA-induced mechanical and thermal hyperalgesia (Zhang et al., 2020a). This rapid postsynaptic mechanism supplies the electrophysiological substrate for central sensitization initiation.

2. p38 MAPK and NF-κB signaling pathways

Running parallel to rapid postsynaptic modulation, CCL2/CCR2 simultaneously fires up long-term inflammatory pathways at the transcriptional level. CCL2/CCR2 activation can trigger the p38 MAPK cascade via the Gβγ subunit. Under peripheral inflammatory conditions, CCL2-driven p38 MAPK phosphorylation subsequently activates NF-κB, promoting Cox-2 and pro-inflammatory cytokine (IL-1β, IL-6, TNF-*α*) expression in the spinal dorsal horn (Lu et al., 2022; Ji and Suter, 2007). Recent work reveals IL-33/ST2 signaling drives macrophages and neutrophils to release CCL2, which then activates the CCR2-TRPV1/TRPM8 axis in DRG sensory neurons, promoting C-fiber-evoked long-term potentiation (LTP) in the spinal cord—unveiling a fresh IL-33-CCL2-TRP channel mechanism in inflammatory pain (Wang et al., 2025).

3. Regulation of presynaptic glutamate release

Beyond postsynaptic actions, CCL2/CCR2 also contributes to central sensitization by controlling presynaptic neurotransmitter release. CCL2 can enhance glutamate release from primary sensory neuron central terminals by activating presynaptic CCR2 (Ma et al., 2020). Ma used the iGluSnFR glutamate sensor together with electrophysiology to show CCL2 acts directly on CCR2 in CGRP-positive sensory nerve terminals in the spinal dorsal horn. By strengthening presynaptic calcium signaling, it increases glutamate release frequency, thereby amplifying NMDA receptor-mediated excitatory postsynaptic currents (eEPSCs) (Ma et al., 2020). This presynaptic mechanism works alongside the postsynaptic ERK-GluN2B pathway, jointly contributing to central sensitization formation and maintenance.

#### 3.2.3 The CCL2/CCR2 axis and neuroimmune interactions

The neuronal regulatory mechanisms described above weave into an interactive network with immune system activation. After peripheral nerve injury, DRG neurons and spinal astrocytes continuously release CCL2, which recruits and activates circulating monocytes/macrophages via CCR2, driving their infiltration into the spinal dorsal horn (Kubíčková et al., 2020). Recent studies have gone further, showing peripheral CCL2-CCR2 signaling is critical for low-dose IL-2-induced regulatory T cell (Treg) migration to the dura mater and trigeminal ganglion—a neuroimmune regulatory mechanism essential for relieving behavioral sensitization in medication-overuse headache (MOH) (Zhang et al., 2020b).

### 3.3 Mechanisms of action of the CX3CL1/CX3CR1 signaling axis in chronic pain

**Figure 2:**
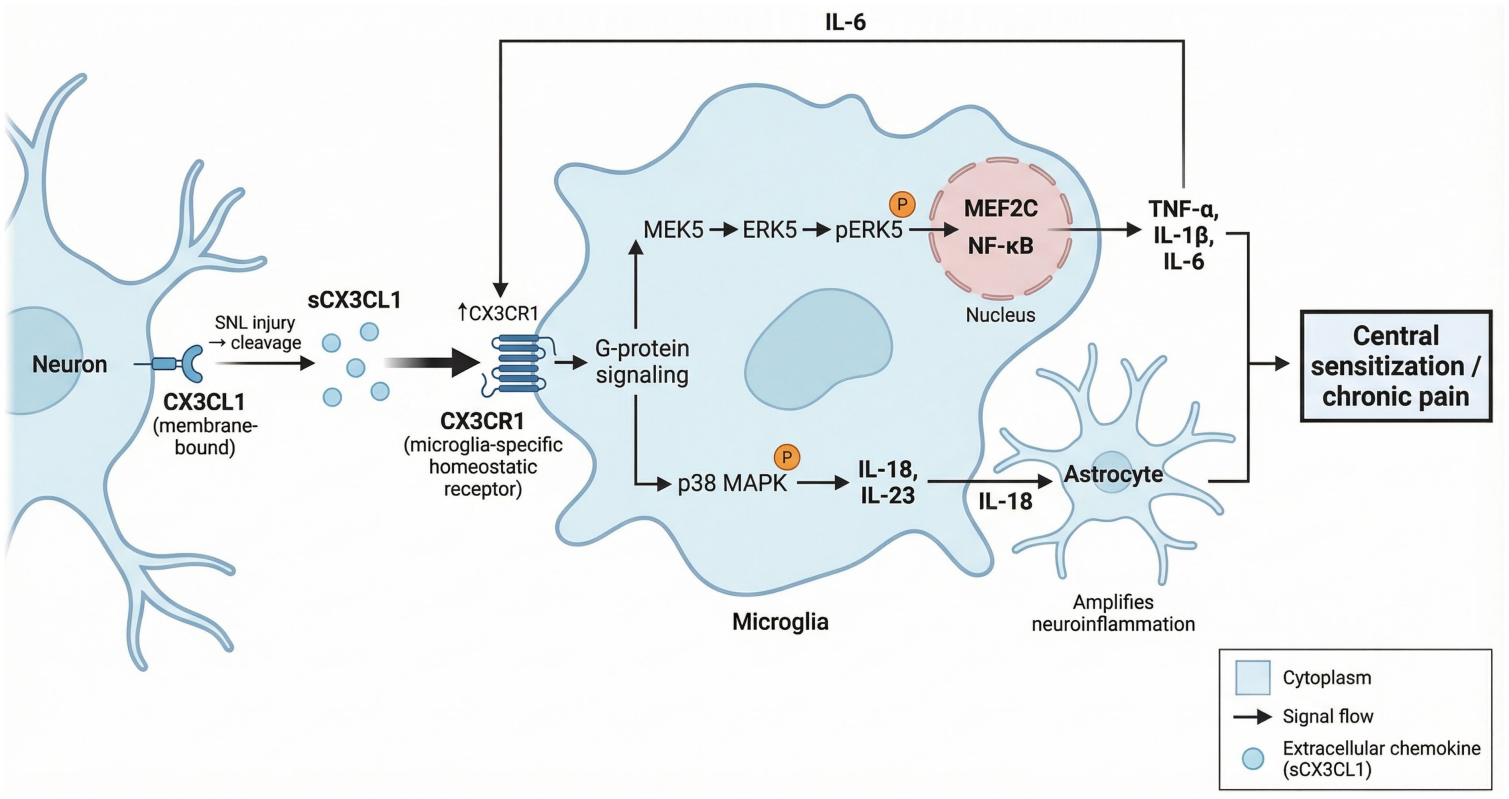
Schematic of CX3CL1/CX3CR1 axis in chronic pain pathogenesis. This diagram illustrates the neuron–microglia crosstalk mechanism mediating central sensitization and chronic pain after spinal nerve ligation (SNL) injury. Following SNL injury, membrane-bound C-X3-C motif chemokine ligand 1 (CX3CL1) on neurons is cleaved to generate soluble CX3CL1 (sCX3CL1). sCX3CL1 binds to the microglia-specific homeostatic receptor C-X3-C chemokine receptor 1 (CX3CR1), upregulating CX3CR1 expression and activating G-protein signaling. This triggers two downstream signaling cascades: one is the MEK5-extracellular signal-regulated kinase 5 (ERK5) pathway, which induces ERK5 phosphorylation, activates transcription factors MEF2C and nuclear factor kappa-B (NF-κB), and promotes the secretion of pro-inflammatory cytokines TNF-α, IL-1β, and IL-6. The other is the p38 mitogen-activated protein kinase (MAPK) pathway, which induces production of IL-18 and IL-23; IL-18 further acts on astrocytes to amplify neuroinflammation. Collectively, these inflammatory mediators drive central sensitization and the development of chronic pain.

Unlike CCL2/CCR2, which mainly mediates neuron-peripheral immune cell interactions, CX3CL1/CX3CR1 represents another core pathway for direct neuron–microglia communication; together they form a complementary neuroimmune regulatory system in chronic pain (Figure 2).

#### 3.3.1 Molecular characteristics of the CX3CL1/CX3CR1 axis

CX3CL1 (fractalkine) is currently the only known CX3C subfamily chemokine, existing in two forms: membrane-bound (anchored via a mucin-like stalk) and soluble (sCX3CL1, released after proteolytic cleavage) (Silva and Malcangio, 2021). CX3CL1’s distinctiveness lies in its constitutive neuronal expression in the CNS, while its sole receptor CX3CR1 is selectively expressed on microglia and serves as a key molecule for maintaining microglial resting state (Noh et al., 2020; Kohno and Tsuda, 2025).

As a microglia-specific homeostatic gene, CX3CR1 preserves immune balance under physiological conditions by regulating microglial motility, phagocytic function, and inflammatory responses (Puntambekar et al., 2022). During inflammation or nerve injury, CX3CR1 expression rises and correlates positively with microglial activation state, making it a functional biomarker of microglial activation in pathological pain (Li et al., 2021).

#### 3.3.2 Downstream signaling cascades mediated by the CX3CL1/CX3CR1 axis in chronic pain

Based on the distribution pattern described above, CX3CL1/CX3CR1 maintains and amplifies neuropathic pain by activating microglia. Its downstream cascades mainly engage two major pathways—ERK5 and p38 MAPK—which regulate long-term inflammatory responses at the transcriptional level and cytokine cascade amplification, respectively.

1. ERK5 signaling pathway

Sun et al. (2013) were first to systematically unpack how CX3CL1/CX3CR1 regulates neuropathic pain through the ERK5 pathway (Sun et al., 2013). After peripheral nerve injury (SNL), ERK5 phosphorylation jumps significantly in spinal dorsal horn and DRG, tracking closely with activation profiles of Iba-1-positive microglia (Obata et al., 2007). Mechanistic studies show that upon CX3CR1 binding, sCX3CL1 activates the MEK5-ERK5 cascade via G protein-coupled signaling. Phosphorylated ERK5 then enters the nucleus, activating transcription factors MEF2C and NF-κB, thereby driving transcription and protein synthesis of pro-inflammatory cytokines (TNF-*α*, IL-1β, IL-6). Intrathecal injection of ERK5 antisense oligonucleotides to specifically knock down ERK5 expression markedly suppresses SNL-induced mechanical and thermal hyperalgesia plus spinal microglial activation, while blocking NF-κB nuclear translocation (Sun et al., 2013). Studies confirm that CX3CR1 gene deletion or neutralizing antibody blockade significantly cuts ERK5 activation after SNL, while ERK5 knockdown also reverses exogenous CX3CL1-induced hyperalgesia, confirming the CX3CL1/CX3CR1-ERK5-NF-κB axis as a central signaling pathway in neuropathic pain onset and progression (Noh et al., 2020).

2. p38 MAPK-IL-18/IL-23 signaling cascade

Parallel to the ERK5 pathway, another major downstream route activated by CX3CL1/CX3CR1 in microglia is p38 MAPK. Zhuang et al. (2007) showed that after spinal nerve ligation (SNL), membrane-bound CX3CL1 on DRG neuron surfaces drops while soluble sCX3CL1 levels in cerebrospinal fluid rise sharply; intrathecal injection of exogenous CX3CL1 rapidly activates p38 MAPK in dorsal horn microglia and induces mechanical allodynia, whereas CX3CR1 neutralizing antibodies can block SNL-induced p38 MAPK activation and pain-related behaviors (Silva and Malcangio, 2021). Current studies indicate the CX3CL1/CX3CR1-p38 MAPK pathway participates in spinal long-term potentiation (LTP) formation and chronic pain maintenance by regulating pro-inflammatory cytokines IL-18 and IL-23 expression (Miyoshi et al., 2008; Hu et al., 2012). Miyoshi et al. (2008) further confirmed that intraspinal IL-18 production is directly controlled by p38 MAPK, and microglia-derived IL-18 can mediate microglia-astrocyte interactions via its receptors, thereby amplifying neuroinflammatory cascades. Recent work also reveals that after peripheral nerve injury, IL-6 induces CX3CR1 upregulation by activating p38 MAPK in microglia, forming a “CX3CL1/CX3CR1-p38 MAPK-IL-6-CX3CR1” positive feedback loop that markedly enhances microglial sensitivity to CX3CL1 and continuously amplifies spinal neuroinflammatory responses (Serizawa et al., 2021).

3. Dynamic regulation of neuron-glial communication

Based on the mechanisms described above, CX3CL1/CX3CR1 mediates dynamic spatiotemporal interactions between neurons and glia during different pain stages. Using an infraorbital nerve ligation (IONL) trigeminal neuralgia model, this study illuminated dynamic features of intercellular communication mediated by CX3CL1/CX3CR1 in the caudal subnucleus (TSC) of the trigeminal spinal tract nucleus (Zhuang et al., 2007):1–3 days post-surgery, CX3CR1 expression in microglia surges, accompanied by increased cathepsin S (CatS) release, suggesting sCX3CL1-mediated neuron–microglia signaling drives early pain onset; 7–14 days post-surgery, reactive astrocytes begin expressing CX3CL1 and CX3CR1, while ADAM17 (a metalloproteinase capable of cleaving and releasing sCX3CL1) expression rises, suggesting CX3CL1/CX3CR1-mediated neuron-astrocyte and astrocyte-astrocyte interactions play a crucial role in pain maintenance.

Within the “gliopathy” framework, Ji et al. (2013) emphasized the dual role of CX3CL1/CX3CR1 in maintaining neuron–microglia homeostasis: under physiological conditions, this signaling keeps microglia resting and promotes surveillance functions; however, under pathological conditions, it transforms into a pathological activation pathway, driving excessive microglial activation and neurotoxic factor release—constituting a central component of glial pathology in chronic pain (Malcangio, 2019).

## 4 Chemokine-mediated chronic pain—inflammation regulatory network

### 4.1 Peripheral nociceptive inputs and primary processing

Orofacial pain signals originate in the trigeminal ganglion (TG); somatic pain signals travel through the dorsal root ganglion (DRG) to the spinal dorsal horn (Gambeta et al., 2020). Anatomically, trigeminal sensory A-*δ* and C fibers enter the pons and descend as the trigeminal-spinal tract, running caudally into the upper cervical spinal cord where they synapse with neurons in the trigeminal-spinal tract nucleus. Cross-talk between satellite glial cells (SGCs) and TG neurons launches central sensitization in head and facial pain, subsequently triggering higher brain region responses (Iwata et al., 2024).

Multiple chemokine axes participate in TG neuron-glial communication. CCL2/CCR2 axis studies have uncovered this pathway’s direct role in orofacial neuropathic pain (Zhang et al., 2012). In a chronic constrictive injury (IONC) model of the infraorbital nerve, TG tissue CCL2 spiked at days 1, 7, and 14 post-injury; blocking CCL2 brought mechanical allodynia down to near-sham levels (Iwasa et al., 2019). CXCL10/CXCR3 data add temporal detail: after partial infraorbital nerve ligation, TG CXCR3 mRNA rose significantly on days 3, 10, and 21, with protein peaking on day 10; CXCL10 mRNA climbed on days 3 and 10, with ligand-receptor co-localization on TUBIII-labeled TG neurons (Ju et al., 2021).

Beyond direct chemokine-receptor actions, recent work shows TG satellite glial cells network extensively through Cx43-mediated gap junctions. Abnormally enhanced coupling drives trigeminal ganglion neuron hyperexcitability, pushing orofacial neuropathic pain forward (Kubo et al., 2026). Peripheral noxious signals reach the spinal dorsal horn via primary sensory neurons—this is the critical hub for pain processing and modulation. Nerve injury or inflammation reshapes the dorsal horn: neurons become more excitable, inhibition drops, glia activate, synapses remodel, and neurotransmitter release changes (Kim et al., 2024; Kim et al., 2011).

At the spinal dorsal horn, CX3CL1 sits on neuronal membranes; CX3CR1 appears only on microglia (Kim et al., 2011; Clark et al., 2013). In peripheral nerve injury and HIV-associated pain models, CX3CR1-deficient mice show reduced hyperalgesia tied to attenuated spinal glial pro-inflammatory responses (Ru et al., 2019; Freria et al., 2017). Dorsal horn CX3CR1 jumps significantly by day 3 post-injury and remains elevated for at least 5 months, maintaining chronic pain through CX3CR1-mediated synaptic pruning (Yousefpour et al., 2025). The CXCL13/CXCR5 axis mediates spinal neuron-astrocyte communication. After peripheral nerve injury, dorsal horn neurons upregulate CXCL13; acting on astrocytic CXCR5, this activates the ERK pathway, driving astrocyte activation and pro-inflammatory cytokine release that further sensitizes neurons (Zhang et al., 2017).

### 4.2 Ascending pathways and cortical emotional coding

After initial processing in the spinal cord and brainstem, pain signals travel through ventrolateral and ventromedial thalamic nuclei to the anterior cingulate cortex (ACC). This limbic structure handles pain’s emotional dimensions—particularly aversion—and emotional regulation (Journée et al., 2023). Recent work shows ACC microglial-neuronal communication through the P2X4R-BDNF–TrkB pathway enhances synaptic plasticity and cortical excitability, helping regulate muscle pain (Liang et al., 2025).

The CXCL13/CXCR5 axis plays a notable role within the ACC. After peripheral nerve injury, both CXCL13 and CXCR5 stay persistently elevated. Knocking down ACC CXCR5 eases neuropathic pain’s negative emotional component, possibly by modulating synaptic transmission in ACC pyramidal neurons (Song et al., 2023; Zheng et al., 2024). When ACC neurons boost CXCL13, it acts on neuronal CXCR5 through autocrine signaling, strengthening glutamatergic transmission and fostering pain-related negative emotions (Wu et al., 2019). Descending facilitation mediated by CXCL1 and downstream cascades amplifies pain sensitization and can even produce mirror pain during acute-to-chronic transition (Li et al., 2022a). This directly links spinal dorsal horn CXCL1/CXCR2 signaling to ACC dysfunction, bridging periphery to cortex.

The CXCL12/CXCR4 axis participates in cortical metabolic pain regulation. Under diabetic conditions, microglial activation drives increased CXCL12 release. CXCL12 acts on CXCR4 receptors on ACC glutamatergic neurons (ACCGlu), producing ACCGlu hyperactivity and diabetic pain. ACC CXCL12/CXCR4 signaling can also boost neuronal excitability through PLC-IP3-Ca^2+^ pathway activation—validated independently in bone cancer pain models (Hu et al., 2017). Liu and Hu noted CXCL12/CXCR4’s role extends past neuropathic and cancer pain into inflammatory pain and even opioid-induced hyperalgesia (OIH) pathophysiology (Chen et al., 2025).

CCL21/CXCR3 studies underscore the ACC’s key role in orofacial chronic pain. In a rat IONL model, ACC CCL21 expression tracked closely with microglial activation; CCL21 rose transiently in sham rats post-surgery, but remained persistently elevated in IONL animals, significantly exceeding sham levels by day 7; intracerebral CCL21 neutralizing antibody injection cut CCL21 levels, inhibited microglial activation, and relieved pain hypersensitivity (Yibo et al., 2021).

### 4.3 Descending regulation and cortico-spinal communication

The ACC participates not only in pain’s emotional encoding but also directly modulates dorsal horn nociceptive transmission through descending pathways, serving as a central node in cortico-spinal regulation (Chen T. et al., 2018). Under electrical stimulation, ACC glutamatergic neuron excitability rises. Through ACC-VTA (ventral tegmental area) projections, this indirectly inhibits dopaminergic neurons, amplifying pain’s emotional component and inducing anxiety (Song et al., 2024). Under the same conditions, the ACC enhances dorsal horn neuron responsiveness to mechanical pain stimuli, demonstrating its central regulatory role in descending modulation (Song et al., 2024). Simultaneously, an activated ACC boosts dorsal horn excitability through descending facilitation, forming a “cortex-spinal cord” positive feedback loop that promotes chronic pain maintenance. More critically, ACC activation drives CX3CR1 expression and p38 MAPK activation in spinal microglia via descending projections (Li et al., 2022b), providing direct evidence for cortex-spinal cord glial interactions and showing chemokine signaling mediates not just local neuroimmune crosstalk but pathological signal transmission across brain regions (Ji et al., 2013).

### 4.4 Integration of hypothalamic homeostasis regulation and chemokine signaling

The hypothalamus controls energy balance, regulating food intake and modulating energy storage and expenditure by integrating humoral, neural, and nutritional signals. Chronic pain tightly links to hypothalamic homeostasis dysfunction, with chemokine signaling playing a crucial mediating role (Le Thuc et al., 2017). Specifically, the CCL2/CCR2 axis acts directly on lateral hypothalamic area MCH-producing neurons, reducing neuronal activity and cutting orexigenic peptide melanocortin (MCH) expression and secretion, leading to reduced food intake and increased energy reserve utilization. Studies confirm causal links between hypothalamic inflammation and dysregulated feeding behavior, potentially driving involuntary weight loss or obesity (Le Thuc et al., 2016).

Within chronic pain’s glial pathology framework, Ji et al. (2013) noted CCL2-mediated hypothalamic neuronal inhibition involves not just direct chemokine-neuron interactions but also neuroinflammatory environmental shifts from microglial activation, further exacerbating homeostatic dysregulation. The CX3CL1/CX3CR1 axis similarly plays a key role in hypothalamic homeostasis imbalance; in diet-induced obesity (DIO) mice, CX3CL1 contributes to early hypothalamic inflammation by recruiting CX3CR1-expressing microglia. CX3CL1 inhibition reduces inflammation, glucose intolerance, and diet-induced fat accumulation (Morari et al., 2014). Ji’s team (Banerjee et al., 2022) further clarified that CX3CL1/CX3CR1 molecular mechanisms in hypothalamic inflammation closely mirror the CX3CL1/CX3CR1-p38 MAPK pathway at the spinal cord level, suggesting chemokine-mediated glial activation shares similar signaling logic across brain regions.

## 5 Summary and outlook

This article provides a systematic review of the core mechanisms of chemokine-mediated glial-neuronal communication in chronic pain, with a particular focus on the CX3CL1/CX3CR1 and CCL2/CCR2 signalling axes. Among these, the CX3CL1/CX3CR1 axis can activate the ERK5-p38 MAPK cascade in spinal microglia, regulate the transcription of pro-inflammatory factors such as IL-18 and IL-23, and sustain the long-term course of neuropathic pain. Based on this, dual-target inhibitors of p38 MAPK/ERK5 can be developed to achieve precise intervention in spinal microglia by exploiting the pathological upregulation of CX3CR1. The CCL2/CCR2 axis enhances glutamate release presynaptically and, postsynaptically, increases NMDA receptor currents via the ERK-GluN2B pathway while also activating NF-κB to promote the transcription of inflammatory genes; this represents a common mechanism underlying both neuropathic and inflammatory pain. Therefore, the development of selective CCR2 small-molecule antagonists can address the issues of poor blood–brain barrier penetration and the difficulty of achieving effective concentrations in the spinal cord through systemic administration. In summary, the mechanism-translational and integrative research presented in this paper will provide new targets and strategies for the precision treatment of chronic pain.

## Glossary

ACC: Anterior Cingulate Cortex
BDNF: Brain-Derived Neurotrophic Factor
CNS: Central Nervous System
DRG: Dorsal Root Ganglion
EMP: Erythro-Myeloid Progenitor
eEPSCs: Excitatory Postsynaptic Currents
GFAP: Glial Fibrillary Acidic Protein
GPCRs: G Protein-Coupled Receptors
IONC: Chronic Constriction Injury of the Infraorbital Nerve
IONL: Infraorbital Nerve Ligation
LTP: Long-Term Potentiation
MCH: Melanin-Concentrating Hormone
MOH: Medication Overuse Headache
OIH: Opioid-Induced Hyperalgesia
PNS: Peripheral Nervous System
SGCs: Satellite Glial Cells
SNL: Spinal Nerve Ligation
TG: Trigeminal Ganglion
Treg: Regulatory T cells
TSC: Spinal Trigeminal Nucleus Caudalis
VTA: Ventral Tegmental Area
